# Communication and Meaning-Making Are Central to Understanding Aesthetic Response in Any Context

**DOI:** 10.3389/fpsyg.2020.00473

**Published:** 2020-03-18

**Authors:** Melissa J. Dolese, Aaron Kozbelt

**Affiliations:** ^1^Department of Psychology, State University of New York at Potsdam, Potsdam, NY, United States; ^2^Department of Psychology, Brooklyn College of the City University of New York, Brooklyn, NY, United States

**Keywords:** aesthetic experience, affordances, communication, meaning-making, Gricean maxims, aesthetic emotions, inclusion, common humanity

Conceptions of aesthetic experience extend beyond beauty to include any evaluative judgment or emotion experienced in response to an artwork. In this opinion piece, we discuss the nature of artistic communication and how it might be facilitated inside and outside of museum settings.

## Aesthetic Affordances and Communication

Many conceptions of aesthetic experience begin with perception. Gibson (1986) argued that an object is perceived in terms of its affordances, which represent potentials for action—like the handle of a coffee mug inviting a grip. Design principles are built around the idea that objects suggest appropriate behaviors (Withagen et al., 2012). Beyond objects' pure functionality, the perception of affordances also involves cognitive, emotional, and aesthetic processes, which emerge via interaction (Xenakis and Arnellos, 2013).

Art objects like paintings do not show physical affordances, like coffee mugs. However, art is a potent source of other kinds of affordances—aesthetic and social—which involve a communicative process and invite a search for meaning. We interact with art objects with the implicit awareness that they were created by other people. As social creatures, our evolutionary survival depended on an ability to communicate and share meaning with others. This habit carries over into our interactions with works of art. Artworks are perceived as extensions of their creators (Newman et al., 2014).

Many factors—including features of artworks, viewers, and physical contexts—impact people's interaction with artworks, both in the lab and in more ecologically valid settings (Pelowski et al., 2017). Empirical studies have also examined more specific facets of artistic communication. These include the extent to which abstract marks can communicate particular emotions (e.g., Takahashi, 1995), the detection of high-quality abstract paintings with reference to perceived artistic intention (Hawley-Dolan and Winner, 2011), and the influence of contextual information—often wall labels in museums—on aesthetic appreciation (Russell, 2003).

## Gricean Principles of Conversation Apply to Visual Artworks

An overarching question is how to characterize the nature of artistic communication. Communication is a meaning-making process, a search for understanding and relevance, and a way of establishing common ground and connecting with another's experience. We argue that principles of communication in everyday conversation also apply to communicative exchanges between artworks and viewers. Specifically, we have proposed (Grice, 1975) maxims of conversation as a promising framework for aesthetic communication in visual art (Dolese et al., 2014).

The Gricean framework is an *intentionalist* model of communication. It presupposes an underlying *cooperative principle*, whereby those involved in an interaction do so with the goal of being understood and arriving at some form of meaning. This plays out via four conversational maxims: *quality* (be truthful), *quantity* (be informative), *relation* (be relevant), and *manner* (be clear). When the maxims are adhered to, or even when they are intentionally unfulfilled (Mooney, 2004), the meaning of a speaker's utterance can be inferred by the listener. When maxims are violated, a speaker is perceived as no longer cooperative, negative emotions arise, and the conversation ends. The Gricean framework is thus useful in characterizing both *direct* and *indirect* communication—that is, not only the choice of words to facilitate straightforward information-sharing, but more broadly in how interlocutors negotiate and develop a shared understanding.

Gricean principles can be translated into the domain of visual art, if one regards aesthetic encounters as a conversation between the artwork-as-extension-of-its-creator and the viewer. For instance, quality can be construed as the artist's sincerity and skill in expression; quantity, as conveying an appropriate level of visual complexity; relation, as a sense of an artwork being relevant to one's experience; manner, as the stylistic and compositional aspects of a work that clearly convey an intended meaning (Dolese et al., 2014).

The divergent experiences of viewers faced with challenging artworks can readily be understood in Gricean terms. Knowledgeable viewers, who share common communicative ground with such works, can easily negotiate deliberate violations of the maxims. For instance, they may appreciate Cubist works that appear to flout the maxim of quality, Minimalist works whose simplicity seems to undercut quantity, or conceptual pieces whose interpretive ambiguity looks as if it would violate manner. In contrast, when inexperienced viewers are confronted by works that violate their expectations, and they have no means of establishing common ground that would allow indirect communication, their response involves negative aesthetic emotions like disgust or hostility (Silvia and Brown, 2007), or, as we note here, alienation—precisely the attitudes that would terminate in-person conversations.

## Implications of the Gricean Framework for Aesthetic Communication

Beyond serving as a mere theoretical model for understanding the nature of artistic communication, a Gricean perspective also has practical implications. Here we focus on three substantive issues where it might provide scientific traction and pragmatic impact: (1) facilitating heightened aesthetic engagement and understanding, especially in museum settings, (2) emphasizing the maxim of *relation* as a means of engaging and empowering viewers, again in museum settings, and (3) better understanding the nature of peak, transformative aesthetic experiences through a Gricean lens.

## Using Gricean Principles to Heighten Aesthetic Engagement

A key element of the Gricean framework is the necessity of shared common ground as a basis for any communication. To the extent that some viewers don't “get” a work or style of art, one might attribute this to missing common ground. This Gricean diagnosis suggests a straightforward remedy: provide additional relevant information, as part of museum walls texts, audio guides, or educational outreach programs that would begin to provide such common ground in an explicit way. Content and stylistic information appears to improve viewers' ratings of the meaningfulness and interest of artworks (Cupchik et al., 1994), though the effect of additional information on aesthetic emotion may be more muted (Dolese and Kacinik, 2019). Thus, information specific to artists' communicative goals, rather than just background content information, may lead to more fulfilling aesthetic experiences.

Establishing common ground becomes more urgent in cases of more challenging artistic styles. For instance, many viewers react negatively to abstract art—even renowned works by famous painters—on the basis, arguably, of not realizing an intentional violation of the quality maxim. However, the finding that even naïve viewers can detect traces of intentionality, which distinguish professionally produced abstract paintings from visually similar works by animals or children (Hawley-Dolan and Winner, 2011), suggests a nucleus of potential common ground that could be developed, particularly in a museum setting.

While such practices could be useful for bootstrapping common ground for aesthetic communication, there may be limits on the usefulness of methods that are *too* explicit. Consider processing fluency, whereby aesthetic response reflects how easily a viewer processes the stimulus of an artwork: easier processing, a more positive response. Hedonic responses appear higher if the source of fluency in processing is unknown to the viewer and the experience comes as a surprise (Reber, 2012). The uneasy relation between explicit knowledge that promotes aesthetic response, and too much information that might potentially dampen it, is a provocative area of research that can mutually inform both Gricean and processing fluency accounts. Clearly, museum contexts would play a major role in future studies addressing these questions.

## The Maxim of Relation Is Crucial for Engaging and Empowering Viewers

The preceding discussion of how Gricean maxims can be deployed in visual art have only used examples from quality, quantity, and manner. We have deliberately withheld a deeper discussion of the maxim of relation until now, as we believe it has special status for informing viewers' perceived representation in settings like museums, which can smack of an elitist ethos.

Viewers often dislike artworks not because of specific objectionable content, but because they seem devoid of meaning. Viewers are thus unable to find a personal connection that makes the work relevant to their concerns and experiences. Indeed, in a series of studies, Landau and colleagues (Landau et al., 2006) found that when forced to confront their mortality, people—especially those with a high need for personal structure—show a decreased liking for modern art that appeared meaningless. Notably, this effect could be offset by imbuing a work with a meaningful title or inducing a personal frame of reference to interpret the work via viewers' own experiences.

In general, viewers have a felt sense of ownership over artworks, an expectation of understanding or meaning-making that should not necessarily require special training or knowledge. This attitude may be compounded by the fact that so much art is found in public venues, which serve to house the memories of the communities it represents: “these institutions show us who we are, who we were, and who we might become” (Smith, 2014, p. 1). Museums have a responsibility to represent the experiences of diverse groups of people and to make clear how these pieces represent individual experience and our common humanity, to honor diversity but also to connect. Visitors frequent museums for information and understanding, meaning, and connection. Those who don't attend often cite a feeling that they don't belong. Art objects can invite movement into institutional spaces by signaling an affordance of belonging, a sense that displayed objects represent visitors, who can thus feel welcomed.

## Meaning-Making Is the Endgame of Aesthetic Response

The process of artistic communication is ultimately geared to the creation of meaning by the viewer. This is a critical aspect of aesthetic response, present in some form in many models of aesthetic appreciation (Pelowski et al., 2016), but whose details and dynamics remain elusive. It is unlikely that artists create work with a very specific point of meaning to communicate—in contrast to someone who, say, creates a visual infographic to represent data. The ambiguity inherent in indirect Gricean communication permits a useful balance between the viewer suspecting that *something* is there to be communicated but then having to work to achieve a meaningful interpretation. Great artworks that communicate indirectly are thus more potent stimuli for aesthetic response and individual meaning-making than any direct, unambiguous communication ever could be.

The process of meaning-making in artistic contexts is not well-understood. Often, it is construed simply as a viewer “getting” a basic understanding of some aspect of a particular artwork—a nice, but decidedly non-peak experience, akin to “mini-c” creativity (Kaufman and Beghetto, 2009) or an aesthetic “experience” tantamount to mild positive affect (Silvia, 2012). Such transient—but not transcendent—moments are not why we care about art.

In contrast to such facile and limited characterizations, other accounts of meaning-making focus on its existential aspects—in confronting and coping with devastating situations of loss, illness, or death (e.g., Frankl, 1946/2006). Meaning-making, in various guises, has sometimes been construed as a central feature of high-level aesthetic response: (Dewey, 1934) view of art as experience (Johnson, 2007), emphasis on embodiment as a vehicle of artistic meaning-making (Konečni, 2005), trinity of peak aesthetic experiences (Vessel et al., 2012), research on intense aesthetic experience and the default mode network, and Pelowski and (Pelowski and Akiba, 2011) discussion of aesthetic experiences that are fundamentally transformative and that can change the way people view themselves.

Articulating the nature of aesthetic communication in more robust, testable terms and with a focus on the endgame of peak aesthetic experience has great potential to inform neglected but vital questions. In-person, museum-based studies of encounters with great art will be necessary to bring this line of inquiry to fruition.

## Conclusion

Understanding how Gricean principles operate should allow us to more effectively engage the process of artistic communication, curating exhibits and environments with the intention to be more relational, communicative, and inclusive, and to spur more intense and meaningful aesthetic responses among viewers. Art is a stimulus for many important modes of human experience: semantic knowledge about culture and the world, aesthetic pleasure in the processing of sensory patterns, interpersonal or social bonding over shared appraisals, and existential meaning-making. Empirically grappling with these themes under the umbrella of artistic communication has extraordinary potential to inform both theoretical and practical aspects of aesthetic engagement, in any context.

## Author Contributions

The conceptual foundations are due mainly to the scholarship of MD, with input from AK. AK and MD each contributed to the writing of this paper.

### Conflict of Interest

The authors declare that the research was conducted in the absence of any commercial or financial relationships that could be construed as a potential conflict of interest.
